# Apraxia of tool use is not a matter of affordances

**DOI:** 10.3389/fnhum.2013.00890

**Published:** 2013-12-20

**Authors:** François Osiurak

**Affiliations:** Laboratoire d'Etude des Mécanismes Cognitifs (EA 3082), Université de LyonLyon, France

**Keywords:** affordance, apraxia, manipulation knowledge, mechanical knowledge, tool use

Until recently, most of our understanding about human tool use has come from left brain-damaged patients, particularly those who show difficulties in actually using familiar tools (hereafter referred to as apraxia of tool use). These difficulties have been suggested to result from impaired sensorimotor knowledge about manipulation (Rothi et al., 1991; Buxbaum, 2001; Buxbaum and Kalénine, 2010; Binkofski and Buxbaum, 2013). The manipulation knowledge hypothesis is very close to the stable affordance hypothesis, that is, the idea that the mere observation of a tool is sufficient to automatically extract stable affordances, namely, invariant features of the tool (i.e., its functional meaning), leading to the activation of the canonical motor action (e.g., Bub et al., 2008; Borghi and Riggio, 2009; Pellicano et al., 2011). In this article, I discuss the viability of the hypothesis that impaired manipulation knowledge/stable affordances might be the core deficit of apraxia of tool use.

Manipulation knowledge is supposed to contain information about the movements associated with the canonical manipulation of a familiar tool (e.g., for a hammer, a broad oscillation of the elbow joint; Buxbaum, 2001). This information is viewed as egocentric, because it specifies the user-tool relationship. On this basis, a parallel has been drawn between the notions of manipulation knowledge and motor affordances (Bub et al., 2008; Borghi and Riggio, 2009; Pellicano et al., 2011). To interact with a tool, some information such as the orientation of the tool has to be processed online, because it does not represent a permanent characteristic. Thus, orientation can be considered as an instance of temporary affordance, processed by the dorso-dorsal stream. Nevertheless, we also have to determine what is the typical orientation of a tool to use it (e.g., the canonical orientation of a book to read it). This typical orientation would be rather based on stable/permanent/canonical affordances, such as shape and size. These stable affordances would involve information stored in memory and might be processed by the ventral, or more particularly, the ventro-dorsal stream (see Borghi and Riggio, 2009; Ferri et al., 2011; Borghi, 2012; Myachykov et al., 2013). In sum, whereas the manipulation knowledge hypothesis focuses on the motor parameters associated with tool manipulation, the stable affordance hypothesis emphasizes the tools' properties useful for a specific manipulation. Whatever, both hypotheses assume that canonical/permanent/stable stored information can be associated with the manipulation of a specific tool, and is a potential basis for the conception of tool actions^1^ (Figure 1).

**Figure 1:**
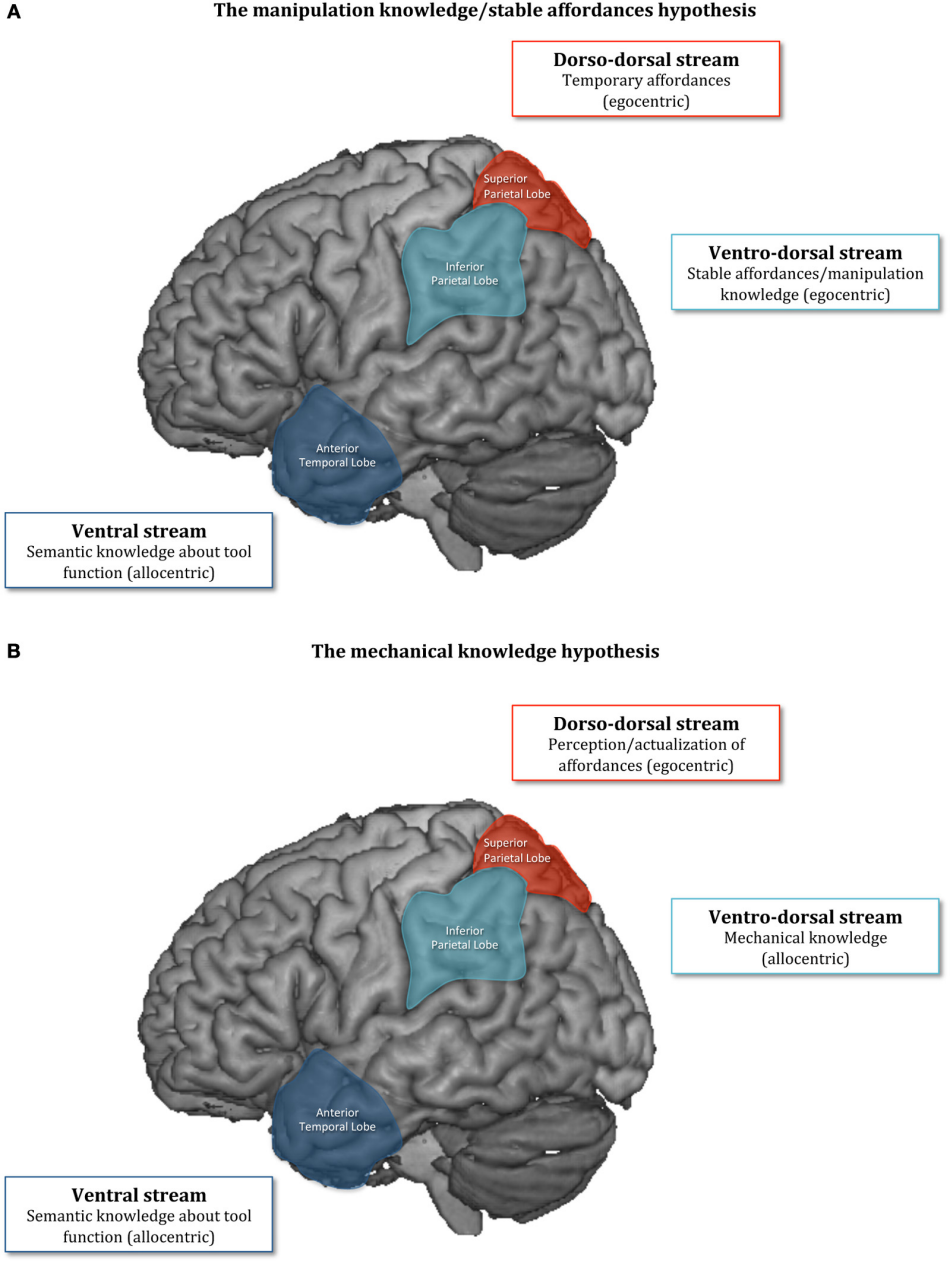
**The manipulation knowledge/stable affordances hypothesis (A) and the mechanical knowledge hypothesis (B)**.

Growing evidence indicates that left brain-damaged patients with apraxia of tool use are impaired to solve mechanical problems, consisting in selecting among several novel tools the one appropriate to lift a cylinder or to extract a target out from a box (Goldenberg and Hagmann, 1998; Goldenberg and Hagmann, 1998; Hartmann et al., 2005; Goldenberg and Spatt, 2009; Jarry et al., 2013; Osiurak et al., 2013; see also Osiurak et al., 2009). These difficulties are associated neither with a dysexecutive syndrome, nor with frontal lobe damage (Goldenberg and Hagmann, 1998; Hartmann et al., 2005; Goldenberg et al., 2007; Jarry et al., 2013; Osiurak et al., 2013). In other words, the ability to use both familiar and novel tools might be supported by a common cognitive process. An important question is whether the manipulation knowledge/stable affordances hypothesis is a good candidate for this common process. At least two theoretical arguments can be offered to conclude that the answer is no.

First, manipulation knowledge and stable affordances are supposed to be associated with a specific tool, more particularly with its canonical manipulation. Given that mechanical problems consist of novel tools, there is no reason that manipulation knowledge supports the solving of these problems. Pellicano et al. (2011) proposed a somewhat more subtle perspective, by assuming that, in some cases, the canonical, familiar tool associated with a usual action can be absent (e.g., to stir coffee in the absence of a spoon). In this case, the usage context might help the user to select among the available tools (e.g., a knife) the one with the most similar structure to the canonical tool (e.g., a spoon). Again, this proposal cannot be applied to the use of novel tools to solve mechanical problems, given that there is no usual context, and no canonical tool associated with the solution of the problem.

Second, manipulation knowledge and stable affordances are thought to be egocentric, in that they specify the relationship between the user and the tool. For instance, Pellicano et al. (2011, p. 1) defined stable affordances as “the potentiation of motor interactions consistent with the conventional use of a perceived tool.” The problem is that, to solve mechanical problems, patients have to form an allocentric representation of the tool solution (e.g., a hooking action involves the relationship between a hook and something that can be hooked). So at a theoretical level, the manipulation knowledge/stable affordances hypothesis cannot explain how this allocentric representation can be formed.

An alternative to the manipulation knowledge/stable affordances hypothesis can be proposed [Osiurak et al., 2010, 2011; Osiurak, 2013; for a somewhat similar view, see Goldenberg (2013)]. This alternative is based on three assumptions. First, when people intend to use tools, the conception of the tool action is not supported by knowledge about the egocentric user-tool relationship. Rather, the conception is based on mechanical knowledge, that is, knowledge about abstract mechanical principles, such as hooking, lever, and percussion. This knowledge is thought to be allocentric, because it specifies the relationship between the different elements of the environment. After all, once people understand the lever principle, they do not need to get a hypothetical, canonical tool to carry out a lever action. Instead, they seek among the different “available” tools, which are immediately within the workspace or not, the one appropriate to the present situation. Said differently, this proposal is the inverse of what Pellicano et al. (2011) suggested: It is not the representation of the stable affordances linked to a canonical tool that guides the search of the appropriate tool; rather, it is the representation of the physical properties useful for achieving the present goal that guides the search of the appropriate tool, whether the canonical tool is within the workspace or not. Interestingly, evidence indicates that the ventro-dorsal stream supports mechanical knowledge (Goldenberg and Spatt, 2009; Goldenberg, 2013). And, impaired mechanical knowledge might be the core deficit of apraxia of tool use.

This leads me to the second assumption: The ability to get appropriate tools that are not within the workspace is supported by what is commonly called semantic knowledge about tool function (Rothi et al., 1991; Buxbaum, 2001; Buxbaum and Kalénine, 2010). This knowledge specifies the purpose, recipient, and context wherein a tool can be used, and is commonly associated to the ventral stream. Evidence indicates that patients with a selective semantic deficit are able to actually use tools, when presented with the corresponding objects (e.g., a hammer with a nail; Buxbaum et al., 1997; Lauro-Grotto et al., 1997; Osiurak et al., 2008; Silveri and Ciccarelli, 2009). However, when the tool is presented in isolation, difficulties can occur, and are strongly linked to the semantic deficit (Sirigu et al., 1991; Hodges et al., 2000; Osiurak et al., 2008; see also Lesourd et al., 2013). In a way, those patients are able to determine through mechanical reasoning how the tools and objects can be used together. However, when tools are presented in isolation, they cannot determine the usual use, because knowledge about the social usages is impaired. Thus, those patients can attempt, on the basis of spared mechanical knowledge, to show that a key can be used for scrapping the chamfered edge of a wooden desk or a nail clipper can be used to attach several sheets of paper together (Sirigu et al., 1991; Osiurak et al., 2008). In other words, semantic knowledge about tool function can be viewed as another form of allocentric knowledge, linking the different tools and objects with the other tools and objects used for the same context or usage (Osiurak et al., 2010, 2011). Thus, when no tool is immediately available (i.e., within the workspace) to carry out an intended action, semantic knowledge can be requested to “mentally travel” over the different semantic categories in search of a potential appropriate tool. In sum, while mechanical knowledge specifies how tools and objects work together to carry out the utilization *per se*, semantic knowledge provides information about the different spaces wherein tools and objects can be found, thereby organizing the search in memory.

The third assumption is that the perception of affordances (and their actualization) only aims to translate the representation of the tool action elaborated through mechanical knowledge (e.g., that the hammer has to make a specific motion to pound a nail) into precise motor programs, linking in an egocentric way the user with the tool. This perspective is consistent with the ecological approach to perception, which assumes that affordances are animal-relative properties of the environment that are not created in the act of perception, but exist independent of it (Gibson, 1979). So people do not systematically or automatically perceive all the affordances that the environment offers to them, but rather only the affordances that are suitable for reaching a current goal [Shaw et al., 1982; Shaw, 2003; Osiurak and Badets, 2014; for a somewhat similar view, see also Tipper et al. (2006), Pellicano et al. (2010), and Ellis et al. (2013)]. In other words, the relevant affordances are *directly* perceived in accordance with the current goal. For instance, among the multitude of affordances that a hammer can offer, people can perceive it as move-able in a vertical plane, when attempting to pound a nail, but they can also perceive it as throw-able when attempting to defend themselves against attackers. Here, the move-ability and throw-ability of the hammer are affordances, but they are perceived only in function of the current goal. In this frame, affordances are necessarily stable, because they correspond to the “negative” of our biomechanical capacities. But, they are also temporary because they are perceived only in function of a specific goal. In sum, there are no stable neither temporary affordances, but only affordances. The corollary is that no affordances about the canonical manipulation of tools can be stored, because they are not engaged in the conception of the tool action *per se*, but are only a way for people to reify the conceptual representation of the action into the physical world. This perspective is much more consistent with the idea that the dorso-dorsal stream is precisely in charge of perceiving and actualizing affordances (see Young, 2006; Figure 1).

To conclude, apraxia of tool use, characterized by conceptual errors in the use of tools, might be not a matter of affordances. Rather, the perception/actualization of affordances would only be involved in the translation of the allocentric, tool action representation into specific, egocentric sensorimotor actions. In fact, in the field of apraxia, the only disorder that might be related to impaired perception/actualization of affordances might be motor apraxia, a disorder affecting the motor coordination mainly of distal movements. Motor apraxia is one of the clinical signs of cortico-basal degeneration (Zadikoff and Lang, 2005). Perhaps, an interesting avenue for future research would be to explore how those patients perceive affordances as usually assessed by the ecological approach to visual perception (e.g., Carello et al., 1989).

## Footnotes

^1^In recent years, a distinction has been made between volumetric/structural vs. functional gestures (e.g., Bub et al., 2008; Buxbaum and Kalénine, 2010; Jax and Buxbaum, 2010). Volumetric/structural gestures correspond to the hand postures used to grasp an object/tool to move it whereas functional gestures correspond to the manipulation of a tool in accordance with its conventional use. This distinction does not fully mirror the stable versus temporary affordances distinction because both volumetric/structural and functional gestures are based on the perception of stable affordances, such as shape or size. Therefore, I will only focus on stable affordances that can be perceived to use tools in a conventional way (i.e., the canonical manipulation of a tool).
